# Supplementary figures and images for: Leveraging plastomes for comparative analysis and phylogenomic inference within Scutellarioideae (Lamiaceae)
Source: PLoS One. 2020 May 7;15(5):e0232602. doi: 10.1371/journal.pone.0232602 (PMC7205251; doi:10.1371/journal.pone.0232602)

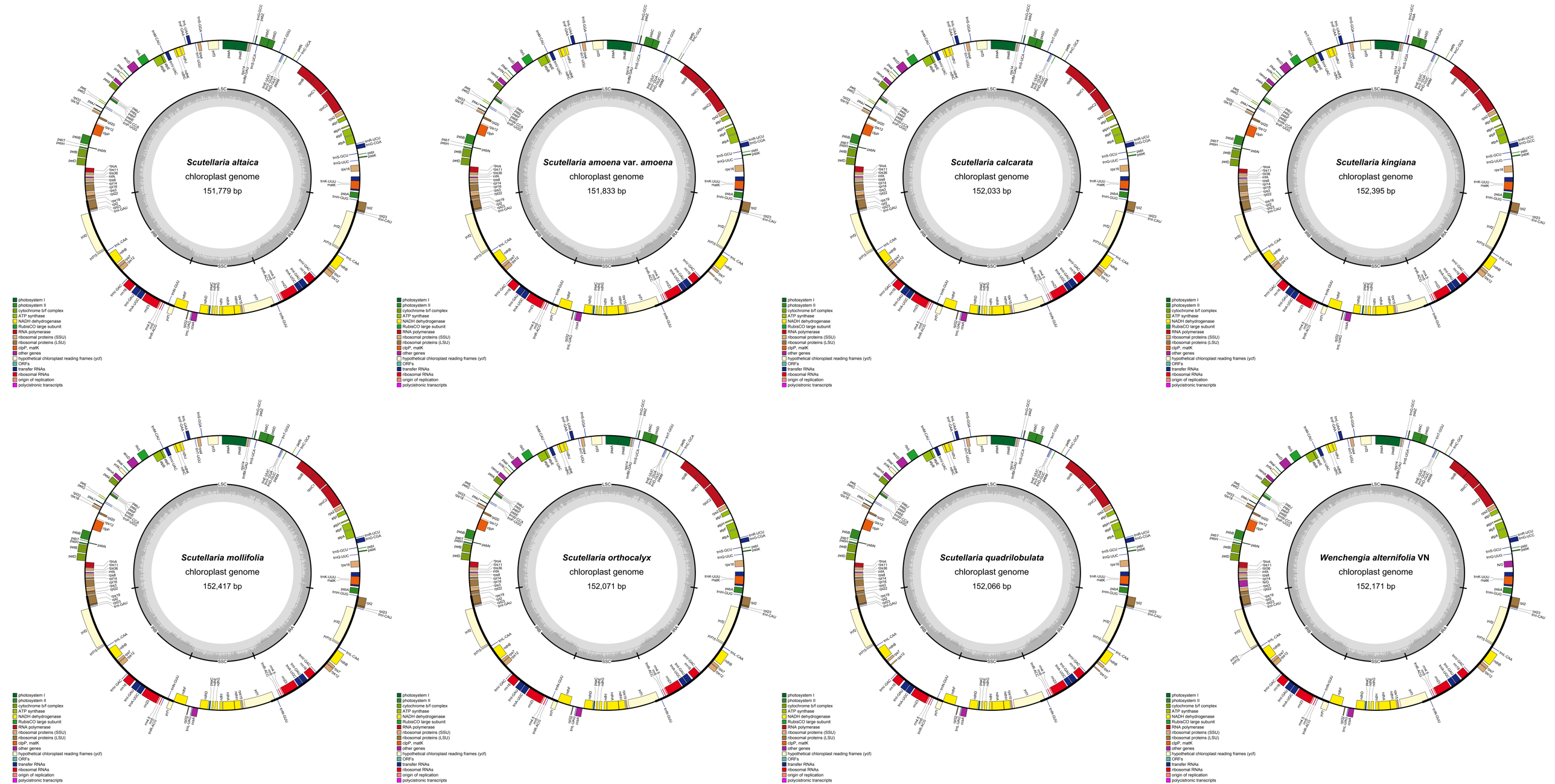

Fig S1

Supplement: S1 Fig — (PDF) [file pone.0232602.s005.pdf]

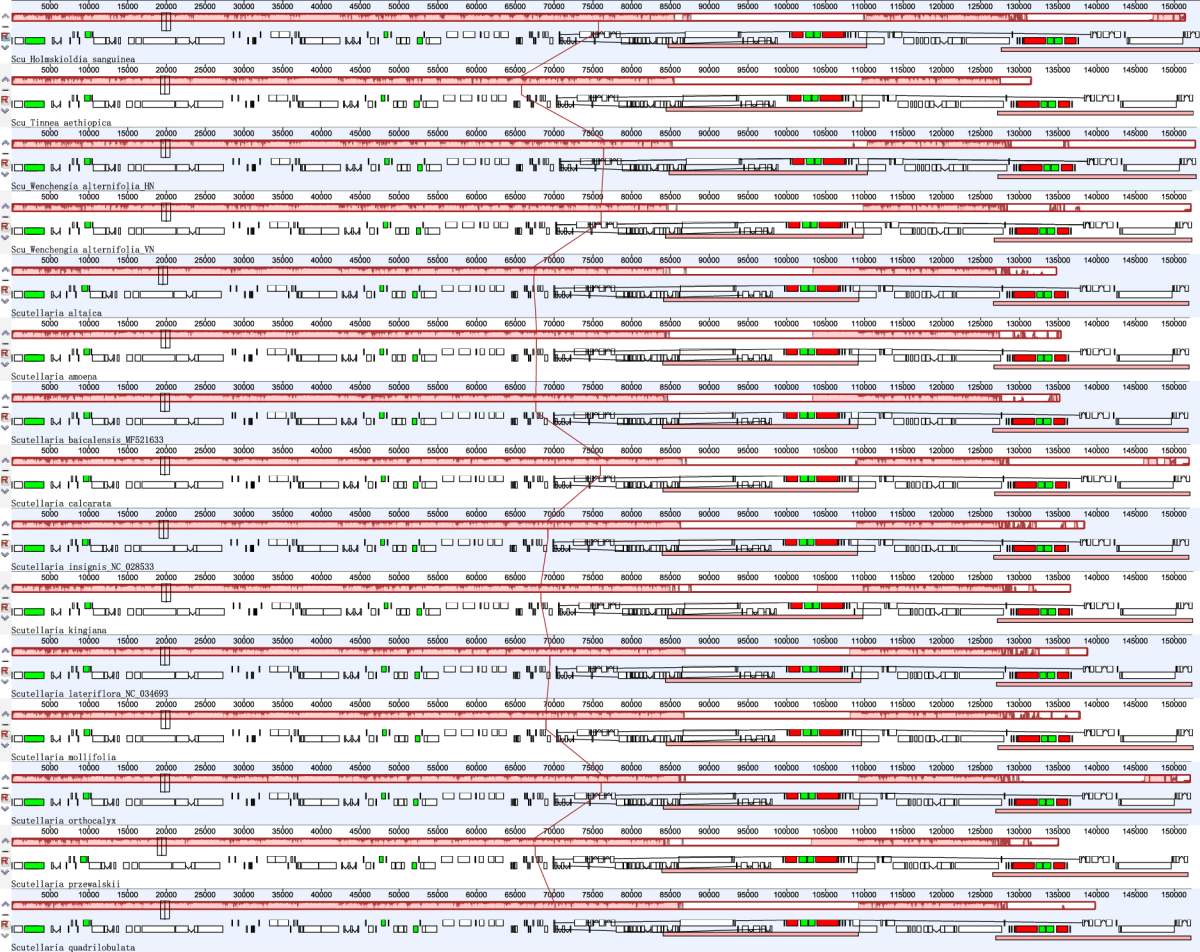

**Fig. S2**

Supplement: S2 Fig — (PDF) [file pone.0232602.s006.pdf]

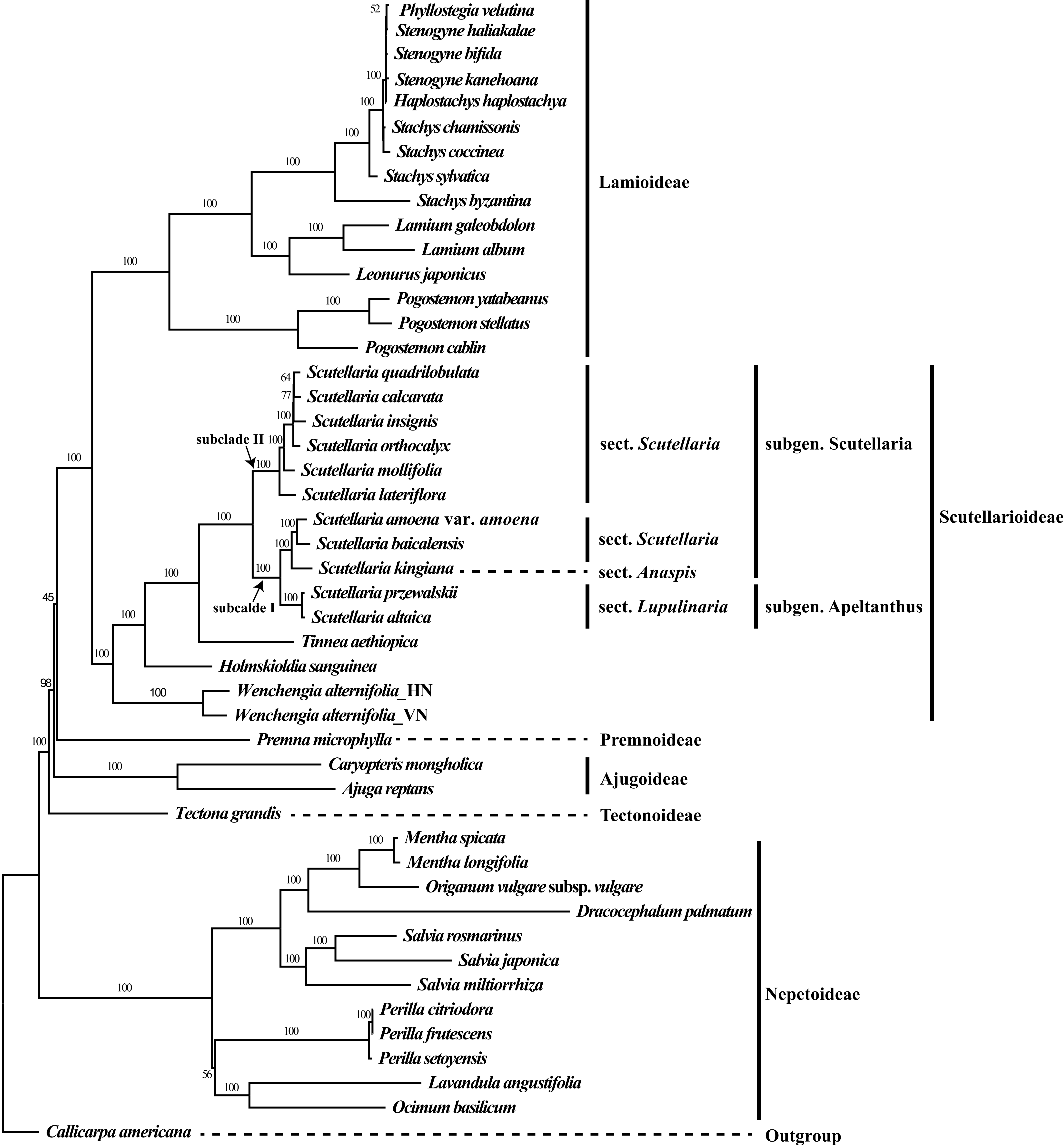

Supplement: S3 Fig — Bootstrap values > 50% are indicated at individual branches. (PDF) [file pone.0232602.s007.pdf]

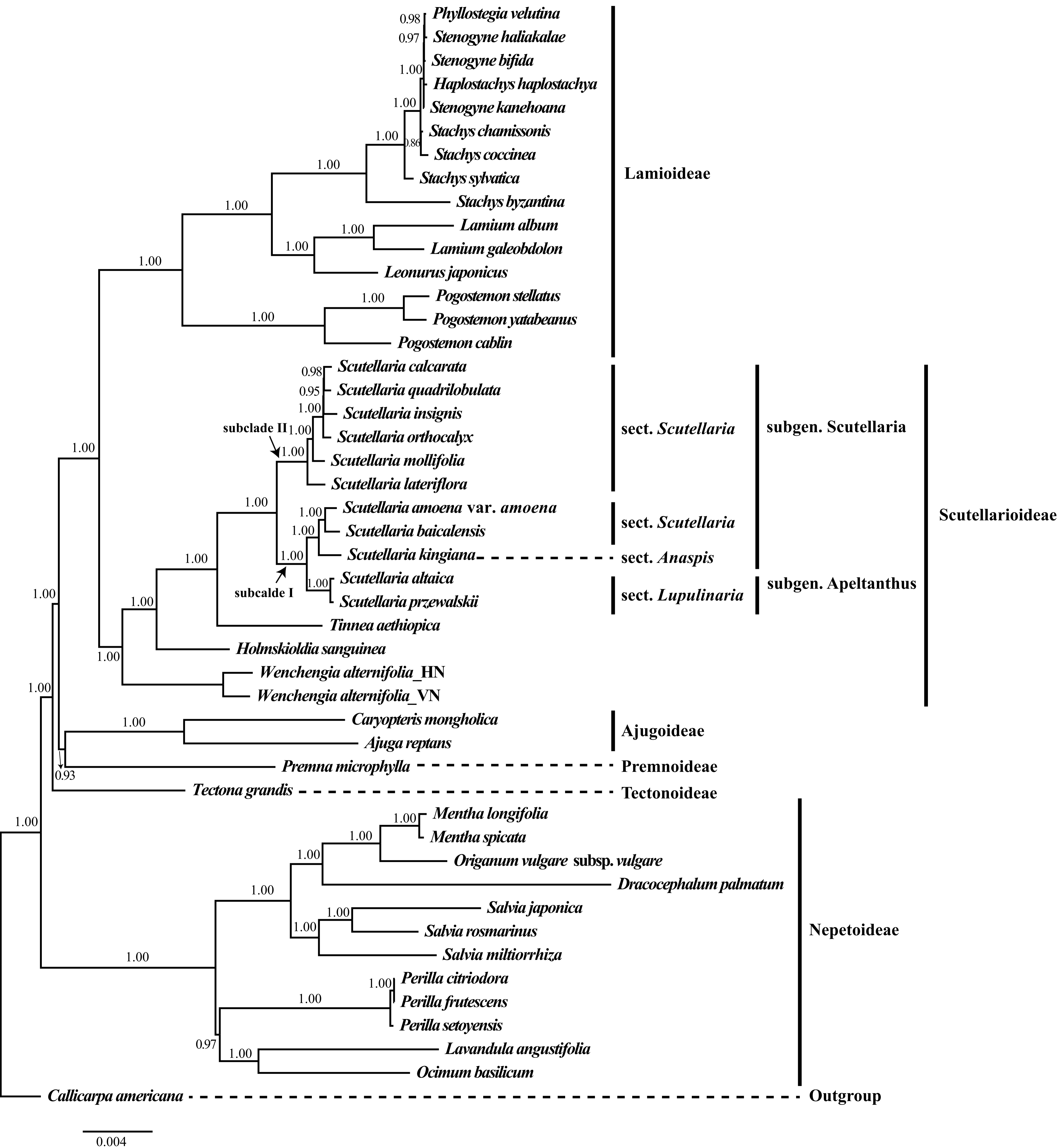

Supplement: S4 Fig — Bayesian posterior probabilities ≥ 0.95 are indicated at individual branches. (PDF) [file pone.0232602.s008.pdf]

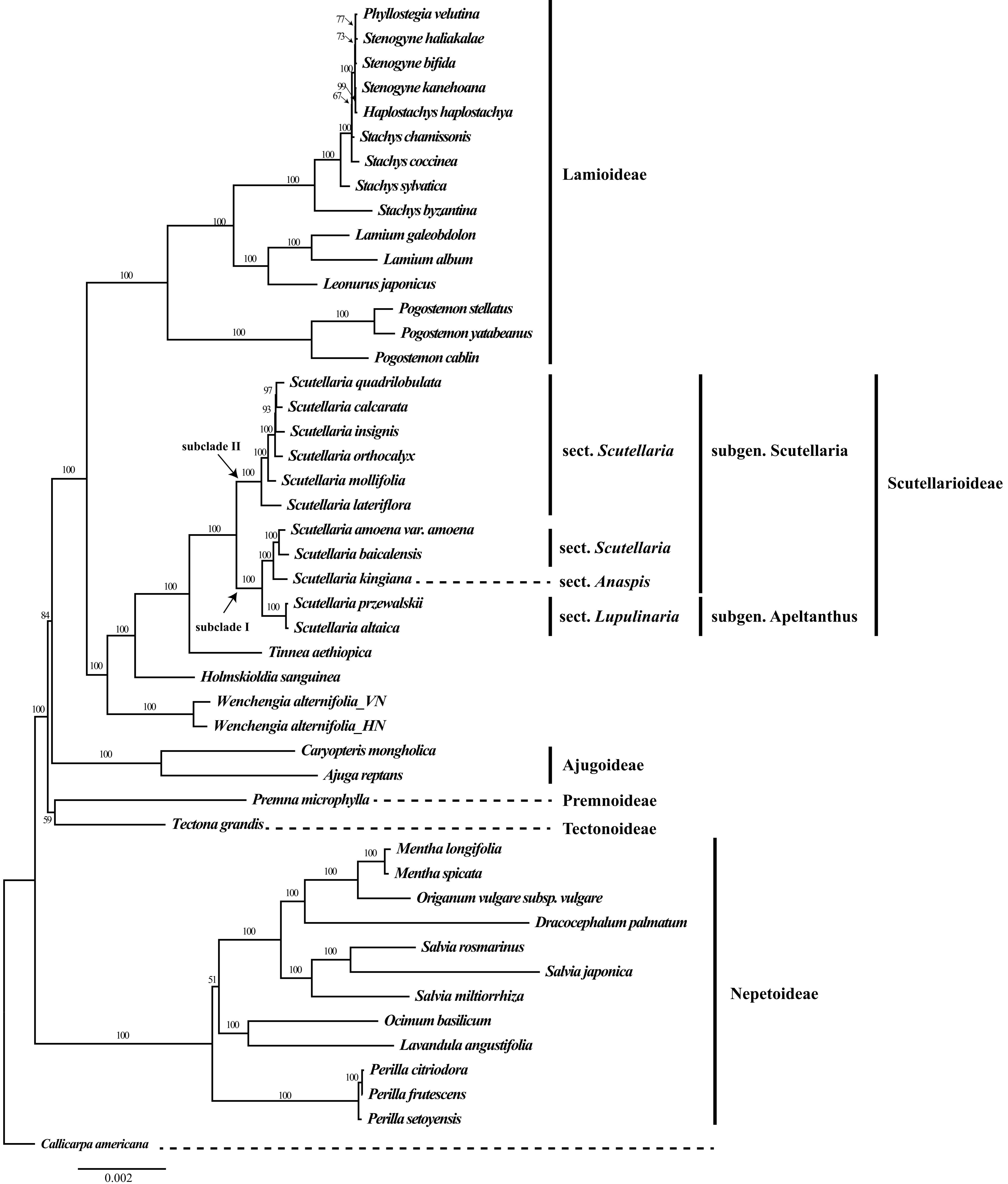

Supplement: S5 Fig — Bootstrap values > 50% are indicated at individual branches. (PDF) [file pone.0232602.s009.pdf]

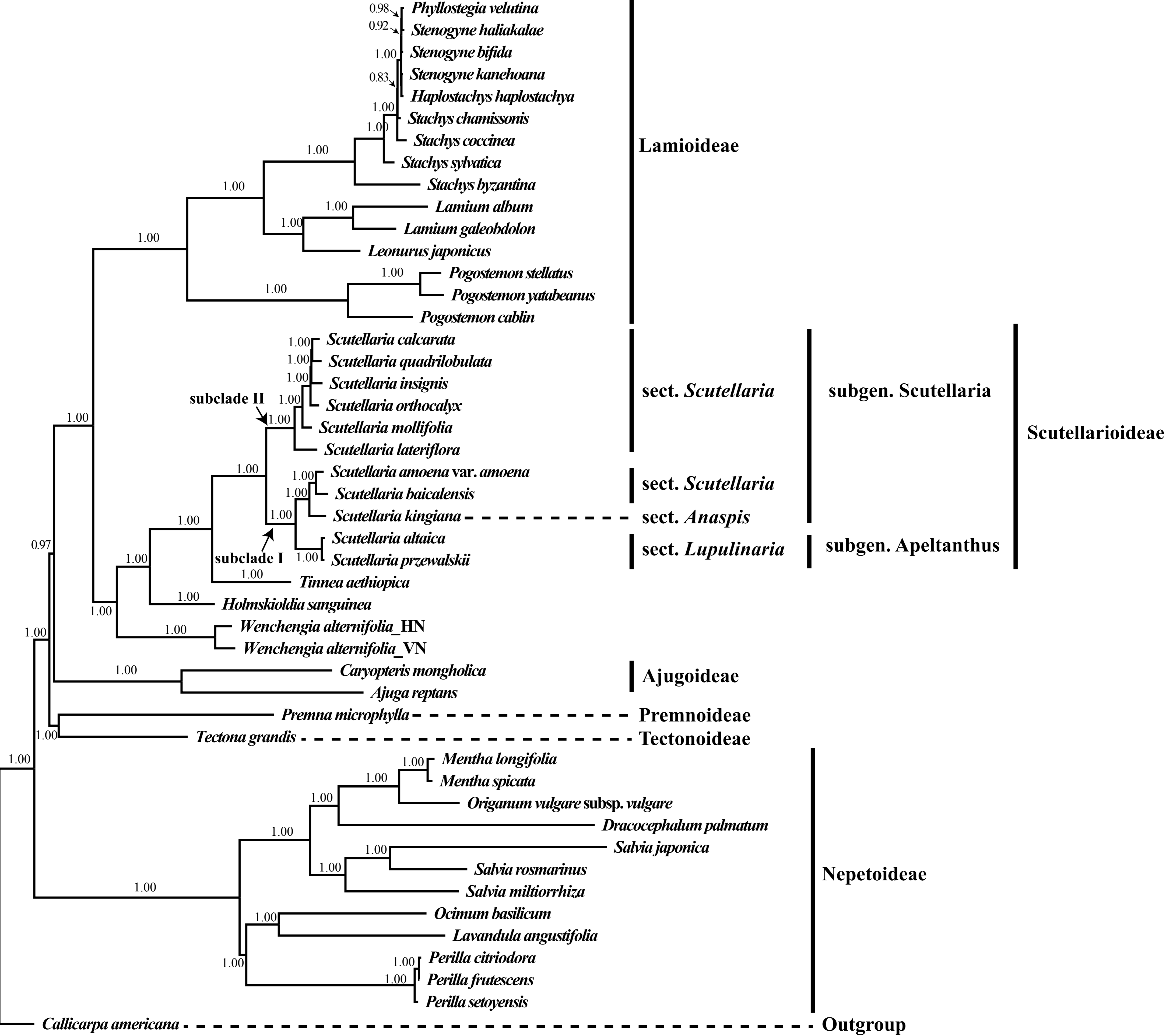

Supplement: S6 Fig — Bayesian posterior probabilities ≥ 0.95 are indicated at individual branches. (PDF) [file pone.0232602.s010.pdf]

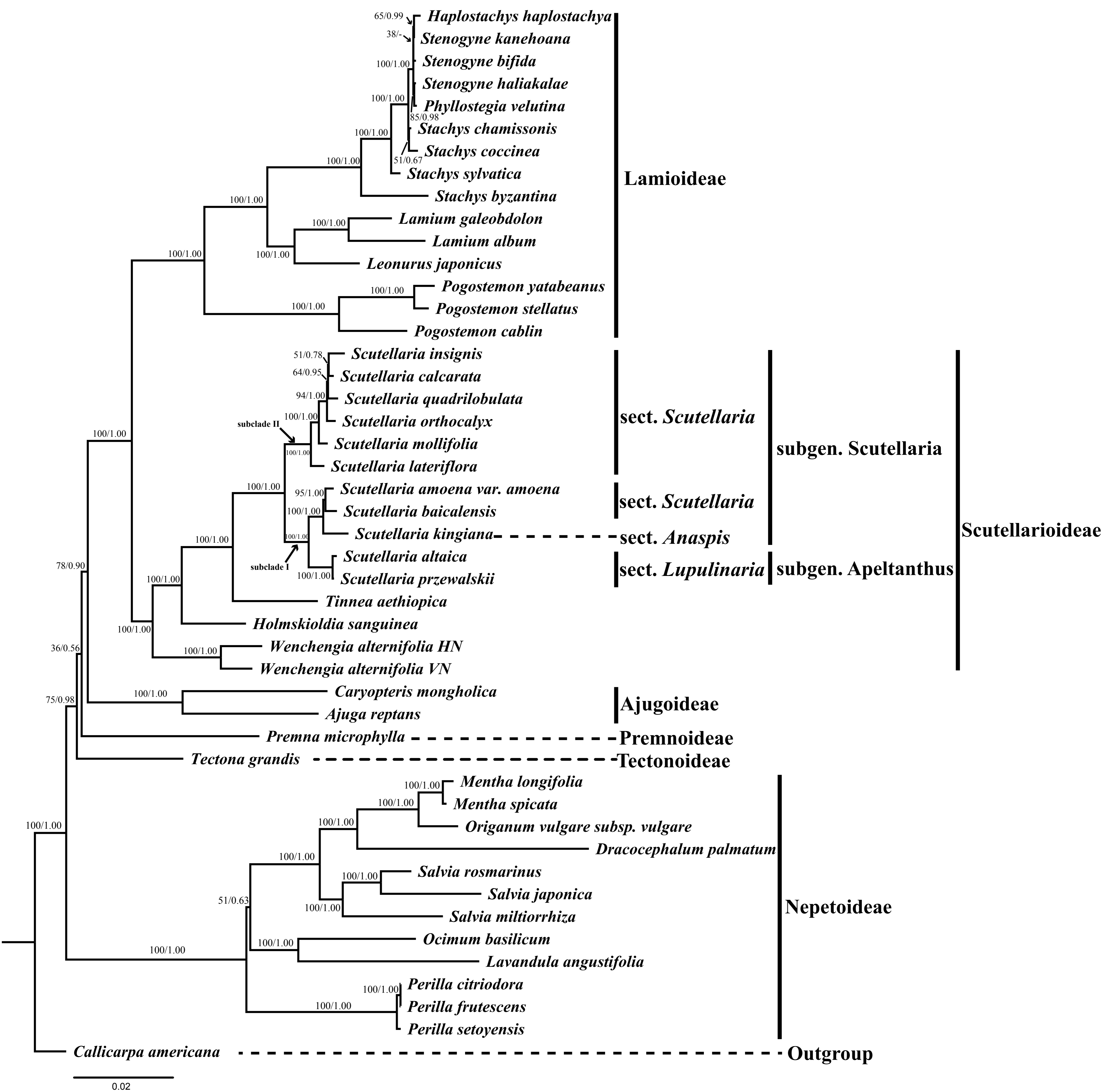

Supplement: S7 Fig — Support values BS ≥ 50% or PP ≥ 0.90 are displayed on the branches follow the order MLBS/BIPP (“-” indicates a support value BS < 50%). Scale bar denotes the expected number of substitutions per site in maximum likelihood analysis. (PDF) [file pone.0232602.s011.pdf]

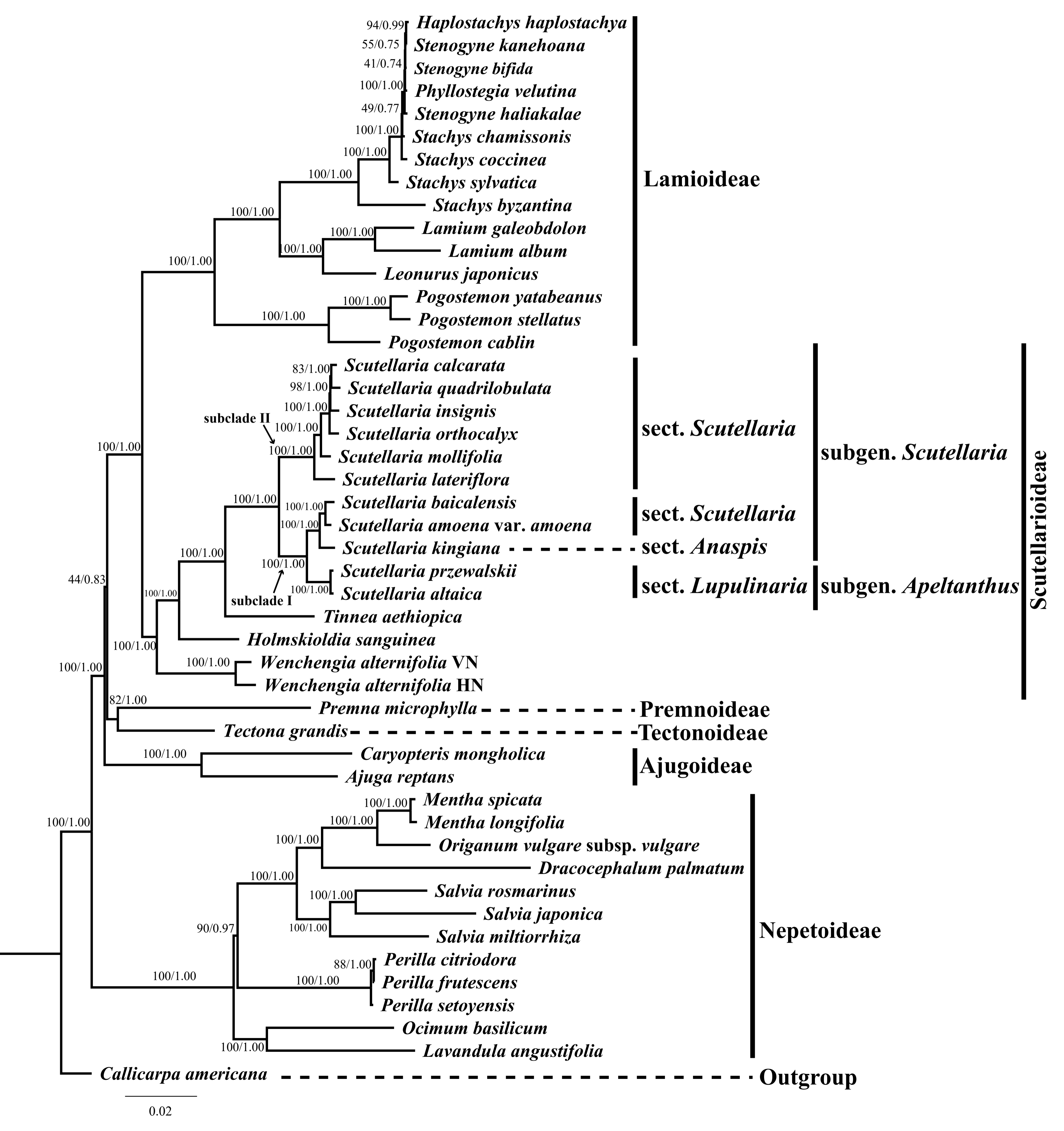

Supplement: S8 Fig — Support values BS ≥ 50% or PP ≥ 0.90 are displayed on the branches follow the order MLBS/BIPP (“-” indicates a support value BS < 50%). Scale bar denotes the expected number of substitutions per site in maximum likelihood analysis. (PDF) [file pone.0232602.s012.pdf]
