# Supplementary material for: Leveraging plastomes for comparative analysis and phylogenomic inference within Scutellarioideae (Lamiaceae)
Source: PLoS One. 2020 May 7;15(5):e0232602. doi: 10.1371/journal.pone.0232602 (PMC7205251; doi:10.1371/journal.pone.0232602)
Supplement: Attachment — Submitted filename: Response to reviewers.docx [file pone.0232602.s013.docx]

**Reviewer 1**

The manuscript by Fei Zhao et al. describes 12 new chloroplast genomes from 11 species and four genera of Scutellarioideae (Lamiaceae). The authors present a careful and thoughtful set of analyses and interpretations, characterizing genome structure and genic content, as well as genomic features and regions with utility for population genetic and phylogenomic studies of the clade. Regarding the latter, they attempt to demonstrate the utility of plastid phylogenomic data for resolving species-level relationships in Scutellarioideae, as well as within Lamiaceae.

I genuinely appreciate the authors’ approach to this study and have no major issues with their methods; most of my comments are intended as helpful suggestions. The pace at which new chloroplast genomes have been generated for Lamiaceae has been relatively slow, and structural variations within the family and the utility of large-scale plastid data in phylogenomic and population genetic studies of mints is not well established. Currently there are only a few published studies that have made these comparisons, and all are clade-specific. Thus, this study represents a new and important contribution to the literature.

I have only a few major concerns that I feel should be addressed by the authors:


1. (L196) The authors made a new chloroplast genome assembly for Callicarpa americana using data deposited in SRA by the Mint Evolutionary Genomics Consortium (2018), but they do not provide any details about this assembly in the manuscript or supplemental tables and have not included an accession ID indicating that the annotated genome assembly is available in an appropriate repository (or at least I am not seeing these details).
Corrected. The complete chloroplast genomes of *Callicarpa americana* were reassembled based on the SRA data (SRR6940059), and thus we didn’t submit it to GenBank and only cited the SRA number in Table S4. Now, we have submitted the complete chloroplast genome of *Callicarpa americana* to GenBank; the accession number is MN883825.

2. It’s not clear from the study results whether complete chloroplast sequence data are necessary to reconstruct relationships in Scutellarioideae or if a smaller matrix of highly variable chloroplast loci (e.g., the list characterized by this study) would also resolve species-level relationships with high support values. This is especially relevant given that sequence capture approaches have very recently become more efficient, reducing off-target reads from the plastome that can be mined and assembled for phylogenomic analyses from targeted capture and high-throughput sequencing of nuclear loci (see de La Harpe et al. 2018); additional financial investments for captures and/or sequencing is now necessary to ensure acquisition of large-scale plastid data for phylogenomic analyses. It is difficult to assess from the data presented here how much plastid data are needed to robustly resolve relationships, especially because comparisons of plastid phylogenomic results are made with published results based on different taxon and DNA sampling schemes. I think this “problem” could be addressed with minimal effort by the authors (see my comment/recommendation for L509 below).

Corrected. Based on the results in the present study, we can say that using complete chloroplast sequence data or matrix of highly variable chloroplast loci are necessary to reconstruct relationships in Scutellarioideae. As suggested by the reviewer, we also selected some commonly used and highly variable chloroplast markers of all sampled taxa in the present study to reconstruct the phylogeny for comparison. Accordingly, more details are provided in the Discussion section.

Additional comments:

L27: It seems rather subjective to refer to Scutellaria as "one of the largest and most taxonomically challenging genera". Please explain.

Corrected. We have rewritten this sentence to minimize the subjectivity (“*Scutellaria* is the second largest and one of the more taxonomically challenging genera within Lamiaceae…”)

L29: This is a picky (semantic) point. A “lack of molecular data” doesn’t directly hinder our understanding of phylogeny. It hinders our ability to accurately and robustly reconstruct phylogenetic relationships, which in turn hampers our understanding of diversity and evolutionary history.

We re-worded this section.

L30-35. The authors should consider alternative phrasing here for improved efficiency.
Corrected.

L50. Pick one: “angiosperm family” or “flowering plants”.
Corrected.

L51. Why is Scutellarioideae one of the most distinctive subfamilies? Explain.
The reason we say that is mostly based on its distinctive morphological characters: the entire-lipped, bilabiate calyx which closed at the mouth in fruit; the corolla zygomorphic, usually 2-lipped or 4-5-lobed; the anterior stamens have unilocular thecae due to the abortion of the upper locule, often referred to as dimidiate stamens; style terminal to sub-terminal, and the nutlets surface frequently tuberculate or bearing long, hair-like processes, attachment-scar usually lateral. The combination of these characters makes this subfamily easily distinguished from other subfamilies of Lamiaceae.

We added “morphologically” to the sentence in question to clarify.

L97. The authors indirectly refer to molecular systematic studies of particular genera, but do not provide citations for these studies as examples. I think it’s fair to do so.
Corrected. we add the relevant reference in the review version.

L105. The authors extensively discuss the need for broad or comprehensive sampling in Scutellaria/Scutellarioideae, and yet this study does not accommodate this need.

Corrected. Main goals of the present study are characterized and compare the structure of plastid genomes within Scutellarioideae and identify candidate molecular makers for future phylogenetic study of Scutellaria. We have prepared another phylogenetic paper for Scutellaria which have more comprehensive sampling. In addition, we have rewritten this sentence.

L159: Please note the correct spelling of “de Bruijn”. What criteria were used in the manual selection of sequence paths (via visualization in Bandage)? This information might be useful to readers.

Corrected. The criteria were mainly based on the coverage and the length of coting.” … the minimum depth of each selected coting 100x and the minimum length > 300 bp.”

L160: Were mapped reads also used to evaluate and report on depth of coverage?
Yes.

L164: DOGMA is an “somewhat dated” tool for chloroplast genome annotation and, although that doesn’t mean it isn’t useful, I’m wondering why the authors chose this tool over newer options. Have they compared their DOGMA annotations with GeSeq (Tillich et al. 2017) annotations?
We use DOGMA for several reasons. Firstly, using the web annotation tool DOGMA can generate a “sequin” file which can be submitted directly to the NCBI. So for the newly sequenced species in the present study, we annotated them using DOGMA. Here, we also used Geseq for chloroplast genome annotation, the results were consistent.

L191–192. This wording isn’t clear. Do you mean that you “evaluated the utility of complete plastome sequences for resolving both the placement of Scutellarioideae in Lamiaceae and species-level relationships in the clade (= traditional subfamily)”?

The author’s use of Lamiaceae-wide sampling seems to go beyond the explicitly stated goals of this study, which focus on Scutellarioideae/Scutellaria (especially without the wording I mention above). Since there isn’t a backbone phylogeny based for Lamiaceae inferred from complete plastome sequences, they may be underselling the significance of their plastid phylogenomic results and its value for the Lamiaceae systematics community. Lamiaceae-wide relationships are only briefly mentioned in the discussion and the importance of the placement of Scutellarioideae in Lamiaceae might be worth highlighting.
Corrected. The main goal of the present study is to evaluate the utility of complete plastome sequences for resolving relationships within Scutellarioideae. The reason we included more Lamiaceae taxa for analyses is because we want to use complete plastome sequences to confirm the placement of the subfamily Scutellarioideae within Lamiaceae. We have prepared another paper for the phylogeny of Lamiaceae based on complete plastome sequences and we will have more details in that paper. So, here we just briefly mentioned the Lamiaceae phylogeny.

L205–206. Which IR copy was used (IRa or IRb)? If IRb was not used, how did you deal with the ycf1 gene that spans the IRb/SSC boundary? And did you remove the rps19 pseudogene?

Corrected. Here only the copy IRa (see Fig. 6) was used for phylogenetic analysis, but both IRa and IRb were included for other analyses. When we prepared the data set, the IRb region was removed and reversed the SSC region. So the ycf1 gene is located in the junction of the IRa and reversed SSC region. The rps19 pseudogene was excluded for phylogenetic analyses.

L244. Previous reports by Jiang et al. (2017) and Lee and Kim (2019) report 114 and 115 genes in chloroplast genomes from Scutellaria sp., respectively. I don’t see any mention of the recent Lee and Kim (2019) manuscript on the S. indica var. coccinea chloroplast genome here. What is the additional (tRNA?) gene? Has it been missed in previous annotations and those included here? Given that new genomes are available since you started your study, it might be worth a quick look and reference in your discussion.

Yes, we have noted their publication. Lee and Kim (2019) reported the complete chloroplast genome of *S. indica* var. *coccinea*, however the sequence of this species was not released (as Jan. 7th, 2020) and those data unavailable to for us for analysis. However, we cited this paper in the discussion.

L405. What do the authors mean by “intense” here?
Corrected. We replaced the “intense” with “extensive”.

L409–411. I appreciate the discussion of types I–IV here. This is interesting. It would be useful to mention any lineage-specific patterns with regard to types observed in Lamiales. (if known by the authors).

Based on previous studies (Chen et al.,2018; Gao et al., 2018), five types were reported from Lamiales. However, there is no close correlation between the distribution of types and linage.

L444. “Though Scutellaria is one of the largest and most important medicinal genera within Lamiaceae”. Is there an appropriate citation for this? This statement seems subjective.

Corrected. We have rewritten this sentence and cited one reference (Irvin, Jackson et al. 2019).

L467. The phylogeny is consistent with studies based primarily on plastid data. However, your results differ from recent nuclear trees (i.e. Mint Evolutionary Genomics Consortium 2018). The incongruence between plastid and nuclear phylogenies emphasizes a need for phylogenetic inferences based on complete plastome sequences, which can robustly resolve relationships (as the authors attempt to demonstrate in this study). Perhaps that is worth mentioning in your discussion.

Corrected.

L509. It is not unreasonable to expect strongly supported relationships in a phylogenetic study with sparse taxon sampling, regardless of whether genome-scale DNA sequence data were used. The authors could easily bolster their argument that use of complete plastome data improves phylogenetic resolution/support if they showed a side-by-side comparison of phylogenetic results, e.g.: (1) phylogeny inferred from commonly used or highly variable chloroplast markers (mentioned in the manuscript) subsampled from their matrix, and (2) your Figure 7 tree inferred from whole chloroplast sequences. This would demonstrate the utility of large-scale data and help avoid making comparisons of phylogenies yielded from studies with different taxon/DNA sampling schemes.

Corrected.

**Reviewer 2_Richard Olmstead**

Review:
This manuscript describes the comparison of 12 new plastid genomes along with 3 previously released plastomes for a total of 15 plastomes of the subfamily Scutellarioideae. The clade consists of the large genus Scutellaria, with 300-400 species and four small genera (three monotypic). Plastome structure, sequence variation, and repeats are presented, along with a phylogenetic tree based on whole genome sequence data.

The presentation is straightforward and mostly descriptive. I have a few more important points, but most of my comments are minor and, I hope, will make the paper a little clearer in places.

Major points.
1. GC content and distribution of SSR and repeats. It has been known for 30 years or more that chloroplast genomes are AT rich, especially in the non-coding regions, where selection is not maintaining GC content for amino acid coding. This paper notes the differences in GC content between large and small single copy regions and the inverted repeats, but doesn’t mention the difference between coding/non-coding regions (Table 4 has GC content for the coding and non-coding datasets, but these are not for complete genomes, if I understand correctly). Separately, the authors note that most of the mono- and dinucleotide repeats are ATs and that they are located primarily in the non-coding regions, but they never put these observations together to conclude that the molecular processes that give rise to repeats (errors in replication) are most likely to be preserved in non-coding regions, because there is strong selection against them in coding regions, and that because the non-coding regions are so AT rich there is an expectation that repeats will be biased towards AT content, especially in the single copy regions. I think it would be great to note this association in the Discussion.

Corrected. The GC content of the complete chloroplast were mentioned in the Table 2.

Yes. Table 4 shows the GC content of the coding region（CR） and non-coding region （NCR）is the modified matrix, not the complete GC content of the CR and NCR, the GC content of the coding region and non-coding region describe in the results “the GC content in the non-coding region (35.0 %) was lower than the coding region (40.5 %)”.

And the reason that the AT repeat content and the location in the non-coding region was expounded upon:

“A potential reason for the higher frequencies of the AT repeats is the strand separation for ATs is relatively easier than GCs during plastome replication, which increases slipped-strand mispairing. There is a tendency for SSRs to occur in the non-coding region of the chloroplast genome of higher plants [82]. The molecular processes that give rise to repeats are more likely to be preserved in non-coding regions because there is strong selection against them in coding regions. In addition, because the non-coding regions are so AT rich, there is an expectation that repeats will be biased towards AT content, especially in the single copy regions.”

2. Counts of SSR and repeat regions in the plastomes are given for individual plastomes and summed for all plastomes (depicted in Figs 2-3), but the aggregate numbers are not very meaningful, because in many, perhaps most, cases these are shared among genomes by descent from a common ancestor. These plastomes represent closely related species that have diverged little in primary sequence, as they show elsewhere in this paper, so the expectation would be that many of these features are shared by descent. This information would be of great interest to people interested in plastome evolution and in the utility of SSR data for evolutionary studies. I would like to see some analysis of the extent to which these repeat elements are shared between related species.

Corrected. a detailed analysis of the shared and unique SSR repeat unit were added in the new version (see Fig. 3).

3. The graphic depictions of the repeat data in Figs. 2 and 3 don’t work very well for me. In my opinion, for example, the Table S2 depicts these data better and more precisely than the bar graphs and pie charts in Fig. 2. This is especially so in fig. 2D, where the many colors are not easily distinguished and the bars are so compressed for all but the A/T mononucleotide repeats that they cannot be interpreted. My point above about repeats shared by common ancestry is important here. By summing the SSRs or repeats in bar graphs like this, the reader is led to believe that there are many independent repeats, when there are likely to be only a few that are shared among many or all sampled species. It would be really interesting to know how many are shared and how widely among Scutellarioideae and how many are unique to individual plastomes.

Corrected. Figure 3 was newly added.

4. I’m a little confused about a few of the Simple Sequence Repeats in Fig. 2D. My understanding of SSRs is incomplete, but I understand that they are repeat motifs of one or a few nucleotides, which exist, of course, in their complement on the opposite strand. Hence repeats like AACC and GGTT count as one repeat (AACC/GGTT in Fig. 2D), because they will exist in exactly equal numbers as complements on opposing strands of DNA and are for all intents and purposes indistinguishable when assessing DNA variation. However, three of the SSRs presented in Fig. 2D and in the text on page 14 are not complement pairs, so I don’t know if I misunderstand their interpretation of SSRs or if there is an error in their results. Specifically, they include the following: AATC/ATTG, AAATC/ATTTG, and AAATAG/ATTTCT.

Results in Fig. 2D (this figure has been deleted in the revised edition; see Fig. 3) were calculated and summarized by the script MISA. Pl. When summarizing the repeat motifs, the repeat of cyclic queues and reverse complements were treated as the same SSR. Although they (AATC/ATTG, AAATC/ATTTG, and AAATAG/ATTTCT) not strictly omplement pairs, they were treated as the same SSR. Here we added one sentence to explain this: When the cyclic queues and reverse complements were regarded as the same SSRs, the 590 SSRs can be classified into 17 different repeat types.

5. IR/SSR boundary presentation. In Fig. 5 and Results, page 16, for Holmskioldia and all of the Scutellaria accessions, the SSC/IRa junction is depicted as falling in the middle of ndhF, while the SSC/IRb junction is depicted as falling in the middle of ycf1. There is something wrong here. Since, by definition the two IR regions are identical, inverted sequence regions, the IR sequence adjoining the two SSC junctions have to be identical. The IR sequence can either be ndhF OR ycf1, but not ndhF on IRa and ycf1 on IRb. If the SSC/IRa junction has moved 25-40 bp into ndhF, then these nucleotides will also appear in the IRb at its SSC junction. If the ycf1 coding frame extends across the SSC/IRb junction, then it would be disrupted by including this sequence in the middle of its reading frame. It is possible that this is the case and that ycf1 is a non-functional pseudogene in Scutellaria (as it is in a number of other plastomes). I think the authors need to look closely at those junctions and sort this out.
In Fig. 5, I think it would be good to show the portion of the ycf1 gene that exists in the SSC end of the IRa, as is done with the rps19 fragment in the IRb at the LSC/IRb junction.
Corrected. we added the annotation label of the pseudogene *ycf1* (marked as *ψ*ycf1) in the Fig.5.and added a description in the text.

Minor comments in order of appearance:

INTRODUCTION
a. P. 2, line 49. Introduction misspelled
Corrected
b. P. 3, line 61. Two early molecular phylogenetic studies that confirmed Cantino’s evidence for the ‘modern’ circumscription I suggest that the one or both of these papers should be cited here to acknowledge that fact. See also p. 22, lines 467.
Wagstaff, S. J. and R. G. Olmstead. 1997. Phylogeny of the Labiatae and Verbenaceae inferred from rbcL sequences. Syst. Bot. 22: 165-179.
Wagstaff, S. J., P. A. Reeves, L. Hickerson, R. E. Spangler, and R. G. Olmstead. 1998. Phylogeny of Labiatae s.l. inferred from cpDNA sequences. Pl. Syst. Evol. 209: 265-274.
Corrected.
c. P. 5, line 113. Plastomes are described as “…multiple copies, and a typically quadripartite…” Add “per cell” after “multiple copies.”
Corrected.
d. METHODS
e. P. 8, lines 175-6. What is “hamming distance?” What was the basis for a minimum repeat size of 30?
The hamming distance in here is that the minimum mismatching characters of two equal-length repeat sequence. The minimum repeat size set as 30bp intend to reduce the time when extended finding the exact repeats (Kurtz et al. 2001). Besides, the parameter set following the published Jiang et al. (2017).

f. RESULTS
g. P. 10 lines 221-2. Describing coverage to 3 decimal places, when coverage varies throughout the plastome seems overly precise. Close enough to take it to the nearest whole intger.
Corrected
h. P. 10, lines 226-7. “The GC content was evenly distributed …” What does this mean? Clearly it is not evenly distributed throughout the genome. I think the authors mean that all of the plastomes in Scutellarioideae have similar overall GC content.
Corrected, Yes I mean that the GC content among the plastomes in Scutellarioideae were same.
i. P. 10, lines 228-9. Please also note the difference between coding and non-coding regions in GC content.
Corrected
j. P. 11, Fig. 1. Figure 5 shows the gene rps19 at the LSC/IRa junction, but this gene is not depicted on any of the circular diagrams in Fig. 1. Is anything else missing?

Corrected. The annotation labels of the ribosomal proteins were missing in the Fig.1 and I re-drown the circular plastome maps of each species of Scutellarioideae and changes the Fig1 in review version.

Also, gene fragments that exist on one IR, because they are part of a gene split by the opposite SC/IR junction (e.g., ycf1) should be identified as pseudogenes with an appropriate symbol.
Corrected. We marked the ycf1 gene fragments as a pseudogene in the Fig 5.
k. P. 11, lines 245-6. “A total of eighteen genes have undergone duplication in the IR region” This should read: “A total of eighteen genes exist in duplicate copies in the IR region.” There is no reason to believe that these have undergone duplication, since the IR is a feature of virtually all plastomes.
Corrected.
l. P. 14, line 275. “The length of repeats ranged from 10 to 139, with an average value of 17 bp.” This is not very meaningful, since a repeat length of 10 was arbitrarily chosen as the lower cutoff for this analysis. Also, I don’t believe the average repeat length is very meaningful, given the fact that many of the repeats will be present in multiple plastomes due to common descent.
Corrected.

m. P. 15, line 294. I would like examples or descriptions of what is meant by forward, reverse, and palindromic repeats.

Based on the repeat analysis software REPuter the Match Direction were defined like this:1. forward (direct) match:

2. reverse match:

3. complement match:

4. palindromic match:

n. P. 16. Lines 325-31. Figure 4. The caption and the legend on the figure need to be aligned better. Does “protein coding” = “exon,” “intron” = “UTR,” etc. I am confused about what is meant by mRNA. mRNA molecules are the product of transcription of protein coding genes and are not, themselves part of a genome. There doesn’t appear to be any gray portions of these plastome figures; does mRNA refer to the arrows above the linear genomes? Why the very long arrow from ca. 99k to ca. 70k labeled rps12? Isn’t this a trans-spliced mRNA and not one very long transcription unit?

Corrected. The legend of the Figure 4 was realigned. In the Figure 4, the “protein coding” = “exon” with the blue color block, the “intron and noncoding region” = “CNS” with the red color block, “trnA gene and rrnA gene” = “utr” with the green color block. The mRNA is refer to the grey arrows above the linear genomes. the very long arrow from Ca. 99k to Ca. 70k was labeled as rps12 was a mistake for the annotation region of the rps12 gene. I didn’t split the two exon of the rps12-5’ and the rps12-3’. While in the new version of the Fig 4 this mistake was corrected.

o. P. 16, lines 332-3. See point #5 above regarding ndhF and ycf1 genes at the SSC/IR junctions.
Corrected. I corrected those in the Fig.5 and in the review version.

p. P. 18, line 375. Caption to Fig. 7. Move the greater-than-or-equal-to sign to be in front of 50%.
Corrected.
q. DISCUSSION
r. P. 19, lines 402-3. “…IR contraction and expansion, has been considered to be a factor underlying species evolution within land plants.” I am unaware of any suggestions that the expansion/contraction of the IR in plastomes has any functional role that may impact speciation or evolution in any way. The cited papers do not mention anything like this. I think the authors mean to say that the variation in IR junction can have phylogenetic signal among relatively closely related species.
Corrected. “The location of the IR boundaries, especially as this pertains to IR contraction and expansion can be exploited for phylogenetic purposes and small expansions or contractions tend to have similar endpoints in closely related species…”
s. P. 19, line 405. “Extensive” instead of “intense”
Corrected.
t. P. 20, lines 428-9. “short dispersed repeats are a major factor promoting plastome rearrangements in land plants, the function of these repeats remains unknown.” Repeat regions have been identified at the end points of inversion in plastomes, but I think most people think this is a random process and that selection acts against most such mutations if they disrupt coding regions or regulatory genomic elements.

Corrected. here my means is that “….short dispersed repeats are a major factor promoting plastome rearrangements in land plants, but within the unrearranged plastid sequences the function of these repeats remains unknown…”
u. P. 20, lines 437-8. “The absence of dispersed repeats from the ycf1 gene in this study may partially explain the sequence conservation of plastomes of Scutellarioideae.” These plastomes are highly similar sequences (not necessarily “conserved”), because they are from closely related species. There may be fewer repeats in ycf1 than in plastomes of some other groups, but I doubt that it is a causative agent for the lack of variation in these plastomes.
Corrected. “…the absence of dispersed repeats from the ycf1 gene may because those plastome sequence from Scutellarioideae were consistent and lack of the variation.”
v. P. 23, line 494. “As shown in Fig. 7, Scutellaria is comprised of two subclades…” Add “in our sampling” to this sentence in front of “Scutellaria.” The sampling is so limited, relative to the diversity of the genus, that this statement is a bit to strong.
Corrected.
w. P. 24, line 521. Fig. S1. What is the zig-zag redline that connects down across the plastomes in this figure?
Corrected. The zig-zag redline indicate correspondence of blocks across genomes.
Signed: Richard Olmstead
