# Supplementary material for: Leveraging plastomes for comparative analysis and phylogenomic inference within Scutellarioideae (Lamiaceae)
Source: PLoS One. 2020 May 7;15(5):e0232602. doi: 10.1371/journal.pone.0232602 (PMC7205251; doi:10.1371/journal.pone.0232602)
Supplement: Attachment — Submitted filename: Response to reviewers.docx [file pone.0232602.s014.docx]

Reviewers' comments:

Reviewer's Responses to Questions

**Comments to the Author**

Reviewer #2: Review of Second submission

Title: Leveraging plastomes for comparative analysis and phylogenomic inference within Scutellarioideae (Lamiaceae).
Authors: F. Zhao et al.
Journal: PLOS One

Review:
In this revision the manuscript is considerably improved and many of the criticisms I raised in my original review are addressed.

Major points:
1) Thanks to the authors for their reply to my query about the difference between forward, reverse, reverse complement, and palindromic repeats. It helped me to see the answer to a different concern I had raised regarding three of the SSRs that are not presented as complements in their paper. These are: AATC/ATTG, AAATC/ATTTG, and AAATAG/ATTTCT. I think these are labeled wrongly, with the mistake possibly being an improper computer output that was not caught by the authors, although I may still be mistaken in my understanding. Let’s work an example.

If AATC/ATTG represents a reverse complement repeat, the reverse complement of AATC would be GATT. In the genome the repeat would look like this: GATTGATTGATTGATTGATTGATTGATT etc.I think an error worked its way into the designation of this SSR by shifting the reverse complement repeat by one nt from GATT to ATTG. Functionally, of course, there is no difference. I think the proper way to present this in text and figures is as AATC/GATT, instead of AATC/ATTG, which does not make sense to me.

A similar adjustment will realign the other two SSRs as well:

AAATC/ATTTG should be AAATC/GATTT (offset by one nt)

AAATAG/ATTTCT should be AAATAG/CTATTT (offset by two nt)

As suggested by the reviewer, we have realigned three types of SSRs in the context, tables, and figures.

2) Figure 3 is a creative way to depict the numbers of different SSRs and how they are distributed among the taxa in the study. However, I don’t think the authors understood my concern about the shared ancestry of SSRs between related taxa The new Fig. 3 shows how many copies of each identified SSR occur is each taxon’s plastome and these are summed across the circle in the individual SSR. However, this doesn’t indicate in any way whether an individual SSR is shared due to common ancestry or not. If the authors have mapped each SSR, they should be able to identify how many unique SSR loci there are. I suspect that there are some that might be shared by all and some that are unique to a single taxon. The number of unique loci will fall somewhere between 48, the maximum number found in one plastome, and 489, the total number of SSRs summed across all plastomes. For my money, this is the most interesting cross-taxon assessment of their data that they could do, but if it is a lot of work to do, I can understand their reluctance to include it.
 Corrected. We provided a new figure to show which SSRs are shared by different species and which SSRs are unique to a specific species.

3) I think there is still a problem with the depiction of the Inverted Repeat/Small Single Copy regions in Fig. 6. At the IRa/SSC boundary, in Holmskioldia and several spp. of Scutellaria, the end of the IRa that is closest to the SSC is shown having both a portio of ycf1 and ndhF. If the IR has migrated into the SSC in these taxa, then I think the ycf1 pseudogene should be offset from the boundary by at least the amount of ndhF that is now found in the Ira. They can’t both occupy the same space. I understand that these are not drawn to scale (e.g., in Holmskioldia there are 1077 nt of ycf1 and only 39 nt of ndhF), but the figure still depicts something that does not make sense. Similarly for those same taxa, the same amount of ndhF will be found in the IRb, but is not depicted there in fig. 6. If ycf1 crosses the IRb/SSC boundary, but the IR boundary has migrated into ndhF, so that a portion (39 bp and perhaps some flanking DNA) of ndhF is now in the IRb, then ycf1 must either be a pseudogene in that location, too, or the ndhF DNA is incorporated into a functional ycf1. The latter is possible, but given that ycf1 is a non-functional pseudogene in some other angiosperms, the likely explanation is that it is so here. Have the authors confirmed that ycf1 is an open reading frame in these plastomes? For that matter, ndhF is known to be a pseudogene in some organisms, too, but I haven’t heard of that being the case in any Lamiaceae, including Holmskioldia, for which an ndhF sequence was determined many years ago.

Corrected. We have modified the location of pseudogene *ycf1* (*ψycf1*) and the *ndhF* gene at the region of IRa/SSC boundary. This modification can show that the *ndhF* gene extended fractionally into the IRa region in some species (i.e., *Holmskioldia sanguinea,* *Scutellaria altaica*, *S. amoena* var. *amoena*, *S.* *baicalensis*, *S*. *calcarata*, *S*. *insignis*, *S*. *kingiana*, *S. lateriflora*, *S*. *mollifolia*, *S*. *orthocalyx*, *S*. *przewalskii*, *S.* *quadrilobulata*)

Minor notes:
P. 14, line 307. Why is this dinucleotide repeat labeled AT/AT, instead of AT/TA? All other repeats are labeled with their complement following the “/”. I think this should follow that convention and be AT/TA here and in any tables or figures.
 Actually, the repeat sequence before and after the “/” show different directions. Sequences before the “/” show a forward reading direction, while sequences after the “/” show a reversed reading direction. So, the repeat “AT/AT” can be explained like this: the AT before the “/” was read in the forward direction, while the AT after the “/” was read in the opposite direction. Then the dinucleotide repeat labeled as “AT/AT”.

P. 14, line 297; p. 15, line 331. On page 14 the total number of SSRs is put at 590; on page 15, it is 489.
 Corrected. On the page 14, the number 590 were the total number of the simple sequence repeats (SSR). While on page 15, the number 489 were the total numbers of the long repeat. In the revised version we corrected it “In total, 489 long repeats including forward, reverse, and palindromic were detected in the 15 plastomes…”

P 14, lines 318-321. Many species names misspelled here.
Corrected. See in the revised.

Fig. 6. Why does sequence identity drop to zero at the 94k mark in the IRa of S. kingiana and also at the 134k mark in the IRb of S. quadrilobata? In both places these are not matched in the corresponding other Inverted Repeat. Since they are in the IR, it doesn’t make sense that these are deletions.
 Corrected. The sequence identity of the 94k mark in the *S. kingiana* and 134k of the *S. quadrilobata* were not zero, but means that percentage of sequence identity below 50% which were calculated by the program mVISTA under the [Shuffle-LAGAN](http://lagan.stanford.edu/lagan_web/index.shtml) model using a glocal alignment strategy. This time, we used LAGAN alignment model to reanalyze, and result were presented in Figure 5.

P. 15, line 356. What does “conserved” mean if the actual boundary varies among species?
 Corrected. Here, “conserved” mean that IRa/SSC boundary within *Holmskioldia* and *Scutellaria* spp. shows a little variation and no structural variation were detected. Now, we corrected it as “……. a small fragment of the ndhF gene extended into the IRa region with (29 bp in *H. sanguinea* and 25–45 bp among species of *Scutellaria*).

P. 15, line 360. “was detected in the IRa/SSC border region.” This is too vague. It is not just in the “border region,” but in the IRa.
 Corrected.

Figure 5. The IR regions are depicted by a line above the rest of the figure. Since the boundary of the IRs vary among taxa, it should be noted in the caption which taxon these regions are specific to.

Corrected. We tried to use a line to show the general location of IR region. We have deleted this line because the boundary of IR regions varied among different species.

P. 22, Line 548. I encourage including the citation for Wagstaff et al., 1998 here, too, since they were first to show Tinnea as sister to Scutellaria (with Holmskioldia sister to them).
 Corrected.

Signed: Richard Olmstead
